# Type 3 gastric neuroendocrine neoplasms: the rising promise of conservative endoscopic management

**DOI:** 10.3389/fmed.2024.1327864

**Published:** 2024-01-31

**Authors:** Elisabetta Dell’Unto, Gianluca Esposito, Maria Rinzivillo, Matteo Marasco, Bruno Annibale, Francesco Panzuto

**Affiliations:** ^1^Department of Medical-Surgical Sciences and Translational Medicine, Sapienza University of Rome, Rome, Italy; ^2^Digestive Disease Unit, Sant’Andrea University Hospital, ENETS Center of Excellence, Rome, Italy; ^3^PhD in Translational Medicine and Oncology, Sapienza University of Rome, Rome, Italy

**Keywords:** gastric carcinoids, neuroendocrine tumors, guidelines, endoscopic resection, management

## Abstract

Gastric neuroendocrine neoplasms (g-NENs) are rare tumors arising from the gastric enterochromaffin-like cells. Recent data suggests an increased detection rate, attributed to more frequent esophagogastroduodenoscopies. While type 3 g-NENs were historically deemed aggressive, emerging research indicates potential for conservative management, especially endoscopic resection, in well-differentiated, small tumors. European guidelines now advocate for endoscopic intervention in selected cases, but North American guidelines remain more conservative. Key factors influencing outcomes are tumor size, grading, and depth of gastric wall infiltration. Endoscopic resection has shown promise for tumors confined to submucosal layers without lymphovascular invasion. Given the complexities, a multidisciplinary team approach is essential for management decisions. Current insights are largely based on retrospective studies, underscoring the need for prospective research to optimize endoscopic approaches.

## 1 Introduction

Gastric neuroendocrine neoplasms (g-NENs) are rare tumors that grow from the gastric enterochromaffin-like cells (ECL cells), with a yearly incidence and prevalence of 0.4 and 3 per 100,000 people, respectively (1). The frequency of these neoplasms has increased in recent years, most likely due to the growing use of routine esophagogastroduodenoscopies (EGD) and the resulting increase in incidental findings (1, 2). As with other NENs, g-NENs can be classified as well-differentiated G1 tumors with a Ki-67 of 3%, well-differentiated G2 tumors with a Ki-67 of 3–20%, and well-differentiated G3 tumors with a Ki-67 of >20%. Gastric neuroendocrine carcinomas (g-NECs) are poorly differentiated tumors with a Ki-67 value greater than 20% (3). Furthermore, g-NENs can be divided into three subgroups based on the presence of an underlying gastric pathology and the presence or absence of hypergastrinemia/ECL hyperplasia: type 1 g-NENs, which account for 75–80% of all g-NENs and are associated with chronic atrophic gastritis (CAG). They are commonly low-grade (G1), well-differentiated, small and multiple lesions, with a risk of metastasis <5% and long-term survival of almost 100%; type 2 g-NEN, which rise in the context of multiple endocrine neoplasia type 1 syndrome, and have an intermediate risk of metastasis ranging from 10 to 30%; sporadic type 3 g-NENs, which rise in the absence of a background pathology, are usually single lesions with larger size (>1 cm) and, compared to other g-NENs, have a greater potential to generate metastasis (up to 50%), resulting in a worse long-term survival (5-year survival rate 70%) (4). Type 3 g-NENs, similar to types 1 and 2, belong to the ECL-cell neuroendocrine tumor family. They predominantly occur in males and are characterized by the absence of hypergastrinemia, and normal oxyntic mucosa surrounding the tumor, which lacks hyperplastic or dysplastic ECL-cell proliferation. The proliferation rates of type 3 g-NENs vary, encompassing low-grade (G1), intermediate-grade (G2), to high-grade (G3) classifications (5). Because the prognosis of these lesions is strictly dependent on tumor type, adequate bioptic sampling of the stomach is required to correctly define the assessment of g-NENs and to properly establish the diagnostic-therapeutic path (4, 6). Traditionally, type 3 g-NENs have been considered aggressive tumors, nearly similar to gastric adenocarcinoma, and thus treated with a radical approach (radical surgery with lymphadenectomy). Nonetheless, in recent years, smaller and more indolent type 3 g-NENs have been incidentally discovered, leading to more conservative care in a subset of patients (4). Because of the increased use of EGD in recent decades, type 3 g-NENs are more usually discovered early, with a reduced size and stage (7). As a result, the scientific community has been debating whether a more conservative method (endoscopic resection) could be feasible in certain patients (8). Different guidelines from Europe and US offer varying recommendations, reflecting the heterogeneity in clinical understanding and approaches to these tumors. This divergence in guidelines underscores the need for harmonized consensus and further research to establish clear management pathways for type 3 g-NENs.

## 2 Search strategy

This review aims to provide insights into the management of type 3 g-NENs, emphasizing the potential significance of endoscopic treatment in their care. Specifically, we will focus on the discrepancies among the various guidelines available for managing these patients. Given the need to regard poorly differentiated NEC as distinct, more aggressive disorders in which endoscopic therapy plays no role, this review concentrates on well-differentiated NETs (thus, the term NET will be used throughout the manuscript). We included data identified by searching the MEDLINE database with no date restriction using the following string of search (“gastric neuroendocrine neoplasms” OR “type 3 gastric neuroendocrine tumors” OR “type 3 gastric carcinoids”) AND (“endoscopy” OR “endoscopic treatment” OR “endoscopic resection” OR “endoscopic management” OR “endoscopic mucosal resection” OR “endoscopic submucosal dissection”). We included only articles deemed relevant to the objectives of this review and written in English. Guidelines from the leading gastroenterology/endoscopy and neuroendocrine tumor scientific societies were consulted.

## 3 Management of type 3 gNETs

### 3.1 Guidelines recommendations

**Figure 1:**
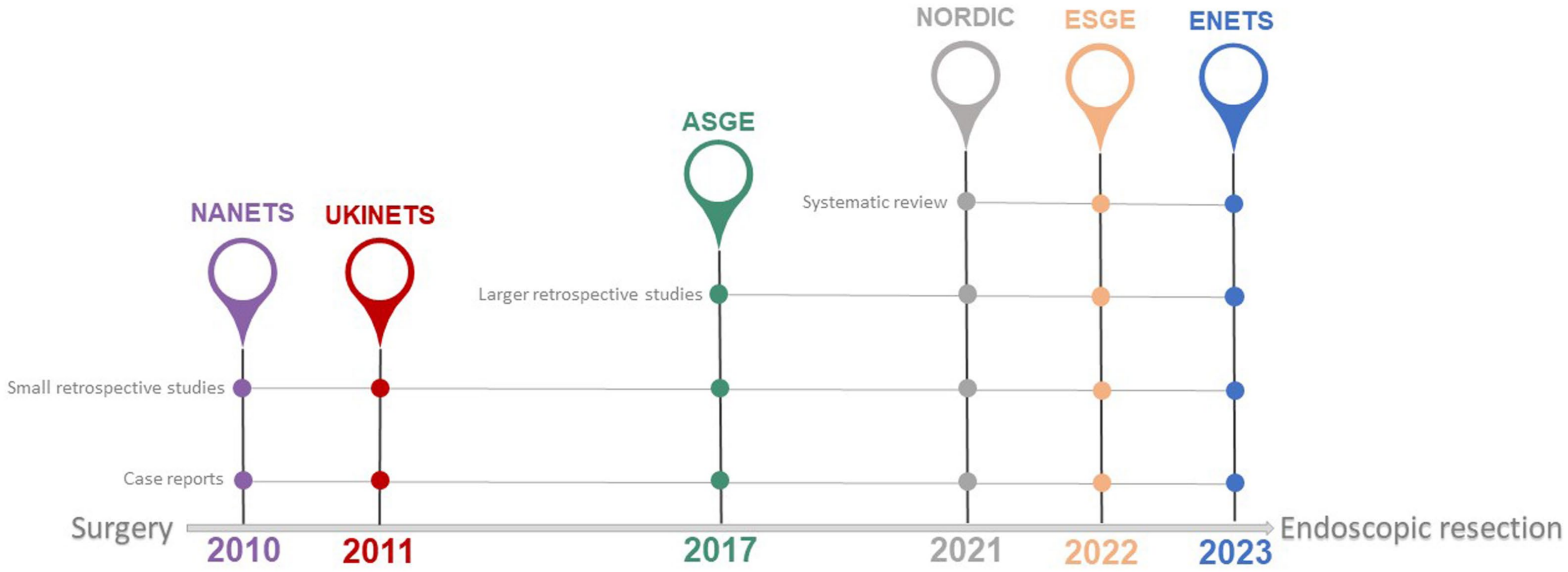
Available international guidelines on management of type 3 gNETs. Year of publication by leading scientific societies involved in the management of type 3 gNETs. A shift toward a more conservative approach is observed from older to newer guidelines (see also Table 1). The continuous grey line indicates the nature of the literature supporting the recommendations in the referenced guidelines. NANETS, North American Neuroendocrine Tumor Society; UKINETS, UK and Ireland Neuroendocrine Tumor Society; ASGE, American Society for Gastrointestinal Endoscopy; NORDIC, Nordic Neuroendocrine Tumor Group; ESGE, European Society of Gastrointestinal Endoscopy; ENETS, European Neuroendocrine Tumor Society.

International guidelines provide heterogeneous recommendations for the management of type 3 gNETs (Table 1). Endoscopic resection of tumors measuring <10 mm and of low grade (G1) is feasible, as per the recent European Neuroendocrine Tumor Society (ENETS) guidance paper, provided metastases are ruled out and the depth of gastric wall invasion is evaluated using endoscopic ultrasonography (4). Also, the European Society of Gastrointestinal Endoscopy (ESGE) suggests that endoscopic resection may be a viable option for type 3 gNETs that are less than 20 mm in diameter, show exclusive submucosal invasion, and have a negative gallium-68 DOTATOC scan beforehand (9). The American Society for Gastrointestinal Endoscopy (ASGE) states that, due to the high likelihood of lymph node metastases, type 3 gNETs should undergo surgical treatment. However, endoscopic excision might be considered for small, well-differentiated tumors (less than 10 mm) (10). Nonetheless, the North American Neuroendocrine Tumors Society (NANETS) guidelines issued in 2010 firmly maintain the indication for extensive surgery for type 3 gNETs (11). NCCN guidelines recommend surgical approaches, such as partial or total gastrectomy (with lymphadenectomy), as the “preferred” method for type 3 gNETs, considering endoscopic resection as an option when EUS or other imaging have ruled out regional lymphadenopathy without specifying indications regarding tumor size (12). Both the Nordic guidelines and the UK guidelines suggest to treat type 3 gNETs in the same way as adenocarcinomas, by performing surgical resection plus lymph node dissection (13, 14). The shift in recommendations toward a more conservative approach in the more recent guidelines (Table 1; Figure 1) is due to recent reports that, although based on small retrospective patient series, have highlighted the potential for safe and effective endoscopic therapy in these patients. The more conservative approach recommended by the ESGE (9) and ENETS (4) guidelines may be due to these guidelines being more recent than others. This underscores the importance of updating guidelines promptly as new scientific evidence becomes available, especially in type 3 gNETs where the scientific evidence is scant.

**Table 1:** Recommendations for treating type 3 gastric NETs according to the different international guidelines.

Year of publication	Source	Recommendation
2010	NANETS	Surgical resection
2011	UKINET	Surgical resection
2017	ASGE	Surgical resection (consider endoscopic resection if tumor size <10 mm)
2021	NORDIC	Surgical resection
2022	ESGE	Consider endoscopic resection if tumor size <20 mm in diameter, exclusive submucosal invasion, negative gallium-68 DOTATOC. Otherwise surgical resection.
2023	NCCN	Surgical resection. Consider endoscopic resection after ruling-out lymphadenopathy (no indication on tumor size)
2023	ENETS	Endoscopic resection in case of G1 tumors, size <10 mm, after ruling-out muscle layer involvement/lymphadenopathy by EUS. Otherwise surgical resection (consider wedge resection as an option).

NANETS, North American Neuroendocrine Tumor Society; UKINETS, UK and Ireland Neuroendocrine Tumor Society; ASGE, American Society for Gastrointestinal Endoscopy; NORDIC, Nordic Neuroendocrine Tumor Group; ESGE, European Society of Gastrointestinal Endoscopy; ENETS, European Neuroendocrine Tumor Society.

### 3.2 Endoscopic approach

Even a few new publications can alter the existing recommendations. Excharcou et al. analyzed 229 individuals with type 3 gNETs in a recent systematic review that comprised 10 nonrandomized retrospective investigations on this subset of tumors. Overall, 51.5% (*n* = 118) of them were well-differentiated G1-G2 NETs, and 121 patients with small and confined lesions received endoscopic excision with Endoscopic Mucosal Resection (EMR) or Endoscopic Submucosal Dissection (ESD) (15). Only one patient, 68 months after the endoscopic resection of a 16 mm G1 NET, had a nodal recurrence during follow-up, implying that selected cases have a better prognosis than previously thought (15). Hirasawa and colleagues investigated similar findings, reporting data on 144 patients with well-differentiated G1-G2 type 3 gNET, 63 of whom had endoscopic resection (48–76.2% - without any other subsequent therapy) (16). Only one patient experienced disease progression during follow-up after receiving conservative endoscopic resection alone, and the 5-year overall survival in the endoscopic group was 100%, with a 5-year recurrence-free survival of 97.6%. It should be highlighted that all type 3 gNETs undergoing endoscopic resection were restricted to the mucosa and submucosa, with a median tumor size of 7 mm, indicating that the best prognostic results are reserved for carefully selected patients (16).

Since an endoscopic strategy to the care of type 3 gNETs had been proposed, several authors pondered what the optimal resection technique was to obtain a complete excision of these lesions. Excharcou et al. reported a rate of complete endoscopic resection (R0) for type 3 gNETs using EMR or ESD of 72–80%, with the highest values obtained using the ESD technique (8); whereas Min et al. obtained a rate of R0 resection in 86.4% of the cases, using primarily ESD but also EMR/modified-EMR (15). However, the optimum endoscopic resection approach in this situation is still debated, despite the fact that ESD appears to be the most successful (17).

### 3.3 Prognostic factors

The identification of prognostic factors capable of predicting disease progression and assisting clinicians in deciding which patient is eligible for endoscopic therapy in those with type 3 gNET is particularly challenging, given the lack of supporting scientific evidence.

Consistent with findings from Excharcou (15) and Hirasawa (16), numerous retrospective studies (8, 18–21) observed positive outcomes following endoscopic resection of well-differentiated, small, low-grade type 3 gNETs limited to the submucosal layers without lymphovascular invasion. Conversely, some studies reported poorer outcomes for type 3 gNETs, often involving cases with larger, higher-grade lesions (22–24). As a result, it is critical to stratify the population based on the presence of unfavorable risk factors, which should guide therapeutic decisions. The most important features that seem to affect the clinical outcomes of patients with a diagnosis of type 3 gNETs, according to a recent systematic review of the literature, are size, grading, and depth of gastric wall infiltration (25). In Hirasawa’s paper, larger G2 lesions with deeper invasion of the gastric wall had a statistically significant higher risk of lymph nodal involvement, with a consequent worse prognosis. The importance of tumor size is highlighted further by the fact that in some cases, extremely small (5 mm) type 3 gNETs were accidentally excised utilizing biopsies and did not recur during follow-up (16). In terms of grading, the Ki67 index is one of the most important risk factors for GEP-NENs in general (26), but its relevance in gNETs is yet unknown (21). In any case, it is prudent to exercise caution before planning an endoscopic resection for a gNET with a high Ki67, given the predictable risk of a more unfavorable biological behavior. Moreover, in this patient setting, there is no evidence to suggest that endoscopic resection is safe. Unsurprisingly, deeper infiltration of the stomach wall with involvement of the muscolaris propria (and beyond) is a risk factor for lymph nodal or distant metastases (18, 24, 27). Endoscopic ultrasound (EUS) is becoming increasingly important in the diagnostic path in this context, to better determine the depth of tumor invasion and lymph node status. Although the role of EUS is well-established in evaluating NETs originating from other parts of the digestive system, such as pancreatic primaries, its role in gastric-originating forms has not been thoroughly studied. The paucity of data on this subject is a major challenge when addressing the scientific literature related to the efficacy of endoscopic treatment of gNETs. Nevertheless, EUS is recommended in patients with type 3 gNETs by the major recent international guidelines, primarily for planning the appropriate mode of endoscopic resection and for conducting a local disease staging to exclude the presence of loco-regional lymph nodes (28).

## 4 Conclusion

Despite being thought of aggressive tumors, type 3 gNETs are increasingly being recognized as more indolent lesions, treatable with conservative endoscopic treatment in selected patients with small, low-grade (G1) lesions. In the absence of standardized selection criteria, such as specific tumor size or Ki67 cut-off levels, it is imperative to evaluate each case individually within a multidisciplinary team discussion (29). Due to the disease’s rarity, there are little data in the literature, primarily from non-randomized retrospective studies with small and widely heterogeneous populations. The guidelines from various scientific societies do not entirely agree on when to use endoscopic resection for type 3 gNETs (Table 1). Specifically, they do not provide clear risk factors to assist clinicians in patient selection. However, there is a trend toward a more conservative endoscopic approach for small tumors (with <1 cm suggested as the cut-off limit, although not standardized), provided that accurate disease staging, including the use of EUS, has been conducted to rule out deep gastric wall invasion and/or lymph node involvement.

## 5 Future directions

There is a pressing need for prospective data to determine the optimal therapeutic algorithm for type 3 gNETs, particularly for small tumors discovered incidentally. As awareness in this specific patient setting increases, there is a growing consensus that traditional surgical approaches may be supplanted by more conservative endoscopic management. Prospective clinical trials are crucial to identify the most effective endoscopic procedure for achieving complete curative resection. Although recent major guidelines uniformly advocate for EUS in disease staging—to both rule out lymph node metastases and assess involvement of the gastric deep layers—no studies have assessed the accuracy of EUS for this particular patient population. Scientific societies focused on NET management should initiate multicenter studies to address these gaps, with the goal of formulating therapeutic recommendations rooted in a robust evidence-based approach.

## Author contributions

ED: Conceptualization, Writing – original draft. GE: Writing – review & editing. MR: Writing – review & editing. MM: Writing – review & editing. BA: Supervision, Writing – review & editing. FP: Conceptualization, Funding acquisition, Supervision, Writing – original draft.
